# Prevalence and social determinants of breastfeeding practices in urban slums and urban non-slum areas in India: A comparative analysis

**DOI:** 10.1371/journal.pone.0323861

**Published:** 2026-04-08

**Authors:** Suliat Fehintola Akinwande, Chiamaka Okonkwo, Kalonde Malama, Thabani Nyoni, Kehinde Oluwatosin Akinwande

**Affiliations:** 1 Factor-Inwentash Faculty of Social Work, University of Toronto, Toronto, Ontario, Canada; 2 State House Medical Centre, Abuja, Federal Capital Territory, Nigeria; 3 Ingram School of Nursing, McGill University, Montreal, Quebec, Canada; 4 Center for Family Health Research in Zambia, Lusaka, Zambia; 5 School of Social Work, Faculty of Health, Dalhousie University, Halifax, Nova Scotia, Canada; 6 Department of Oral and Maxillofacial Surgery, Lagos University Teaching Hospital, Lagos, Nigeria; India Health Action Trust (IHAT), Uttar Pradesh Technical Support Unit (UP-TSU), INDIA

## Abstract

Understanding how the prevalence and determinants of breastfeeding practices differ between urban slum areas, where living conditions are stringent, and urban non-slum areas, which have relatively more resources, is key to context-specific interventions. We conducted a comparative analysis to investigate the prevalence of breastfeeding practices in the urban slum and urban non-slum areas of India. We also used the socio-ecological framework to assess the individual, community, and policy-level correlates of breastfeeding practices. Secondary analysis of data from the National Family and Health Survey (2015–2016) in India was conducted to estimate the prevalence of early breastfeeding initiation and exclusive breastfeeding of children living in urban slum and urban non-slum areas, and the prevalence estimates were further stratified by the seven states where slums were sampled. Multilevel logistic regression analysis was used to examine the correlates of breastfeeding practices. Early breastfeeding initiation was significantly higher in the urban slum areas (50.4%) compared to urban non-slum areas (37.4%). In contrast, exclusive breastfeeding was lower in urban slums (50.1%) than in urban non-slum areas (55.8%). At the individual level in urban slum areas, preceding birth of more than 24 months was associated with early initiation of breastfeeding. At the policy level, child delivery at the health facilities was associated with early initiation of breastfeeding in the urban non-slum areas. The study showed that breastfeeding practices need to be urgently addressed in both the urban slum and urban non-slum areas, and policy-level factors such as health care facilities should be considered in designing effective interventions.

## Introduction

Optimal breastfeeding practices, including early breastfeeding initiation (EBI) and exclusive breastfeeding (EBF), have been recommended as key to reducing child mortality and could save over 820,000 children yearly [1]. EBI within an hour of birth and EBF for six months are fundamental to the health and development of children [2]. The breast produces colostrum, a special milk secreted in the first two to three days of childbirth; it is rich in white blood cells, antibodies, protein, minerals, and fat-soluble vitamins (A, E and K) [3]. Breastmilk contains all the required nutrients and immunity for children in the first six months of life [3]. Exclusive breastfeeding helps prevent diarrhea and infections [4], supports intellectual and motor development, and reduces the risk of chronic diseases [5]. Randomized control trials from low and middle-income countries showed that 72% of diarrhea admissions and 57% of respiratory infections were prevented by breastfeeding [4]. Breastfeeding has also been reported to prevent infectious diseases, mortality from necrotizing enterocolitis and sudden infant death syndrome [3].

Despite these well-established benefits, achieving optimal breastfeeding practices is often more challenging in resource-constrained settings like the slum areas. The environment is characterized by poor sanitation, overcrowding, inadequate ventilation, lack of potable water, and rapid spread of diseases [6], all of which can undermine breastfeeding practices. In addition, mothers living in urban slum areas are more likely to experience social and structural barriers that hinder breastfeeding initiation and continuation [7,8]. The World Health Organization (WHO) reports that more than 1 in 4 deaths of children aged under 5 years occur in urban slum settlements [9]. Therefore, it is important to understand the specific contexts and factors that shape breastfeeding practices in these settings.

Of the 1.1 billion people living in the urban slum areas globally, India accounts for approximately 65.49 million individuals and 13.9 million households living in such under-resourced environments [10,11]. With projections estimating that the urban slum population in India will rise to 105.7 million, and given evidence that suboptimal breastfeeding practices may be exacerbated in these settings, there remains limited research examining breastfeeding practices in urban slum areas [12]. Among the few studies assessing EBF in urban slum areas in India, the application of the WHO guidelines for measuring EBF among 0–5-month-olds is inconsistent; while one study used the recommended age range of 0–5 months, others included infants up to 6 months, thereby raising concerns regarding the validity and comparability of their findings [13–15]. Moreover, studies are missing a comparative aspect to contrast breastfeeding practices in the urban slum and urban non-slum areas, which could help inform best practices for both contexts. Further, studies have mainly looked at individual-level factors (e.g., child’s birth order, mother’s age, and the educational level of the mother) and not addressed the wider determinants of breastfeeding practices in the community and policy levels [13–15]

We designed a study to address the limited evidence base to inform context-specific interventions for breastfeeding programmes in urban slum and urban non-slum areas in India. Our study aims to:

1) Compare the prevalence of breastfeeding practices in the urban slums and urban non-slum areas in seven states in India
2) Identify the determinants of breastfeeding practices at the individual, community, and societal levels across the seven states in India

Hypotheses:

1. H1: The prevalence of optimal breastfeeding practices (including early initiation and exclusive breastfeeding) is significantly lower in urban slum areas compared to urban non-slum areas across the seven states in India.
2. H2: Individual-level factors, community-level factors, and societal-level factors are significantly associated with breastfeeding practices among mothers in the seven states in India.

**Theoretical framework** Based on the socio-ecological model, individual, community, and policy-level characteristics have interlinked effects on breastfeeding practices [16]. The individual-level factors relate directly to the mother and child characteristics, like the child’s sex, birth order, and the mother’s level of education [16]. Due to the prevalent patriarchal system in India, gender differences in the nutrition of children exist, with girls being the most disadvantaged [17]. Specifically, the patriarchal system limits girls’ educational opportunities, which reduces their ability to access and understand key health information once they become mothers [18]. Community-level factors influencing nutrition include sociocultural norms and religion [19]. Studies have shown that women in India from disadvantaged strata/castes and lower socio-economic classes often experience different socio-cultural factors that influence their well-being and that of their child during childbirth, and these practices vary from region to region [20,21]. Such practices include discarding colostrum and isolating mother and child at birth [22]. At the policy level, slum dwellers have been historically marginalized and denied access to health services, which makes mothers in slum areas unlikely to access quality antenatal care during childbirth and postpartum [6].

## Methods

### Data source and sampling

Secondary analysis was conducted using the fourth National Family and Health Survey (NFHS) data of India (2015–2016) [24]. The Ministry of Health in India and the International Institute of Population Science in Mumbai coordinated the NFHS survey, which takes place every five years. NFHS-4 sampling was designed to include urban slum areas in seven states in India (Maharashtra, Tamil Nadu, Uttar Pradesh, West Bengal, Telangana, Madhya Pradesh, and Delhi). The NFHS-4 employed a stratified two-stage sampling design. The 2011 Census served as the sampling frame for selecting primary sampling units (PSUs), defined as villages in rural areas and Census Enumeration Blocks (CEBs) in urban areas. PSUs with fewer than 40 households were merged with the nearest PSU. In each rural stratum, villages were selected from the sampling frame using probability proportional to size (PPS). To enhance representativeness, each stratum was further divided into six roughly equal substrata, formed by crossing three substrata based on the estimated number of households per village with two substrata based on the proportion of the population belonging to scheduled castes and scheduled tribes (SCs/STs). Urban slums were identified using the following criteria: “ 1) a compact area of at least 300 population or about 60-70 households of poorly built congested tenements, in an unhygienic environment usually with inadequate infrastructure and lacking in proper sanitary and drinking water facilities’‘ The following were criteria used to identify slums in NFHS-4; “1) all specified areas notified as ‘slum’ by state/local government and union territory (UT) administration under any act; 2) all areas recognized as ‘slum’ by state/local government and UT Administration which may not have been formally notified as slum under any act; 3) a compact area of at least 300 population or about 60-70 households of poorly built congested tenements, in unhygienic environment usually with inadequate infrastructure and lacking in proper sanitary and drinking water facilities”. The different areas within the cities were classified as “slum” and “non-slum” area respectively [23]. Further details on the survey sampling method have been described elsewhere [24].

### Study population

Women residing in the above mentioned states were interviewed by trained data collection staff using standardized questionnaires based on the WHO format (document in S1 file). Information on feeding practices of the youngest children under the age of 2 years was collected from the mothers using a 24-hour recall, except for EBI, which was assessed using mothers’ responses on the timing of breastfeeding initiation after delivery. We included women aged 15–49 years and their last-born children living with them to reduce the potential impacts of recall bias and erroneous information.

### Outcome variables: Breastfeeding practice indicators

The breastfeeding practices were assessed using the WHO core guidelines [25]. EBI was defined as the proportion of children born in the last 24 months who were breastfed within one hour of birth. EBF under 6 months was the proportion of infants aged 0–5 months who were fed only with breast milk, including medications.

### Explanatory variables

The individual-, community-, and policy-level variables were selected based on previous studies and data collected using the NFHS questionnaire [13–15,26]. The individual-level variables were the child’s sex, child’s birth order (1^st^, 2^nd^-4^th^, ≥ 5^th^), previous birth interval (<24 and ≥ 24 months), age of mother (15–19, 20–34, and ≥35years), religion (Hinduism, Muslim or others), education level of the father and mother (no education, primary, secondary and above), household wealth index, number of antenatal clinics visits (1–2, 3–6, and ≥7), if mother listens to radio, mother reads newspapers, or mother watches television (not at all/at least once a week or almost every day). Community-level variables were class (upper class, scheduled castes, scheduled tribes, and other backward class (henceforth referred to as ‘marginalized class’), and state of residence. The policy-level variable was the place of child delivery (health facility or non-health facility). In the survey, the household wealth index was divided into five quintiles (poorest, poorer, middle, richer, and richest) based on principal component analysis. The index scores were derived from household ownership of consumer goods (e.g., television, bicycle, cars) and household characteristics such as drinking water, floor material and toilet facilities [24].

### Ethical consideration

Our study utilized publicly available data from the NFHS 2015–2016 survey, which contains no identifiable participant information and is accessible via the Demography Health Survey (DHS) website. The Institutional Review Board of the International Institute for Population Sciences (IIPS), Mumbai, and the ICF Institutional Review Board granted ethical approval for the survey. Before each interview, written informed consent was obtained from all participants during data collection. The NFHS-4 dataset is fully anonymized, ensuring that individual respondents cannot be identified. We accessed the datasets with permission from the DHS Program on the 29^th^ of July 2022. Since the data were de-identified and accessed following established protocols, separate ethical approval was not required, and data confidentiality was maintained throughout the analysis.

### Statistical analysis

Descriptive analyses were conducted to determine frequencies and proportions of all variables. The prevalence of each breastfeeding practice (EBI and EBF) stratified by urban slum and urban non-slum residence was estimated and reported with 95% confidence intervals. EBI and EBF rates for each state were also estimated separately for urban slum and urban non-slum areas. Differences in the prevalence of EBI and EBF between both areas were assessed using the Chi-square test, with statistical significance set at p < 0.05.

Multilevel logistic regression was used to examine the relationship between breastfeeding practices and individual-, community-, and policy-level characteristics. Bivariate analyses were first conducted to identify variables for inclusion in the multivariable models, using a p-value <0.20 as the cut-off. A less stringent significance level was selected at this stage to avoid excluding potential confounders and variables that may become important in the presence of other covariates in the multivariable model. This approach is commonly used as a purposeful selection strategy in multivariable modelling [27]. In the final multivariable models, p-values <0.05 were considered statistically significant.

Guided by the socio-ecological model, variables were entered into the regression analysis in three levels—individual, community, and policy—to reflect their hypothesized pathways of influence on breastfeeding practices. Individual-level variables (e.g., maternal education, age, ANC visits, and birth order) were treated as potential confounders, as they could influence both exposure and breastfeeding outcomes. Community-level variables (e.g., caste, religion, and regional factors) were conceptualized as contextual determinants rather than mediators, given their influence on social norms and access to resources. Policy-level variables (e.g., slum residence) were considered structural determinants reflecting access to health services. No variables were treated as effect modifiers in this analysis, although potential interactions were explored descriptively.

Because respondents were nested within primary sampling units (PSUs), a multilevel logistic regression model with random intercepts at the PSU level was fitted to account for data clustering. Sampling weights were applied to adjust for the complex survey design of the NFHS using probability weights (v005). Multicollinearity was assessed using Variance Inflation Factors (VIF). Since VIF cannot be directly computed after multilevel logistic regression, an ordinary least squares (OLS) model with the same independent variables was estimated to obtain VIF values. A threshold of VIF > 10 was used to indicate problematic multicollinearity. We used mixed-effects logistic regression with random intercepts at the cluster level (v021) to account for the hierarchical structure of the data. The intraclass correlation coefficient (ICC) was calculated to quantify the proportion of total variance in the outcome attributable to differences between clusters. The ICC was computed from the variance of the random intercept using the standard latent variable approach for logistic models. An ICC close to zero indicates little clustering effect, whereas higher ICC values indicate substantial between-cluster variation.”

Missing data were assessed for all variables. Mothers’ occupation had over 80% missing values and was therefore excluded from the analysis. Sensitivity checks comparing respondents with and without occupation data showed no significant differences in key sociodemographic characteristics (maternal age, education, and household wealth index), suggesting minimal bias due to this exclusion. Data analysis was performed using Stata Version 15.0.

## Result

### Socio-demographic characteristics of the study

The sample size consisted of 933 and 2315 mother-child pairs in the urban slum and urban non-slum areas, respectively (Table 1). The median age of the children in both areas was 11 months (interquartile range (IQR): 6–17 months). While the median age of mothers in the urban slum was 25 years old (IQR = 23–28) and 26 years old (IQR = 23–29) in the urban non-slum areas.

**Table 1:** Individual-, community-, and policy level characteristics of the study participants in the urban-slum and non-slum areas in India using DHS Data for India, 2015-2016.

		Urban slum areas (N = 933)	Urban non-slum areas (N = 2315)
		n (%)	n (%)
Individual-level	Sex		
	Male children	473 (50.7)	1208 (52.1)
	Female children	460 (49.3)	1107 (47.8)
	Children’s age (months)		
	< 6	165 (17.7)	521 (22.5)
	6- < 24	768 (82.3)	1794 (77.5)
	Birth order		
	1^st^	415 (44.4)	950 (41.0)
	2^nd^-4^th^	499 (53.5)	1301 (56.2)
	5^th^ and above	19 (2.1)	64 (2.7)
	Mother’s age (years)		
	15-19	59 (6.3)	39 (1.7)
	20-34	831 (89.1)	2123 (91.7)
	>=35	43 (4.6)	153 (6.6)
	Mother’s Education		
	No education	174 (18.7)	283 (12.2)
	Primary school	117 (12.6)	241 (10.4)
	Secondary and above	640 (68.7)	1791 (77.4)
	Father’s Education		
	No education	736 (78.9)	2030 (87.7)
	Primary school	30 (3.3)	26 (1.1)
	Secondary and above	166 (17.8)	259 (11.2)
	Antenatal care visits		
	None	62 (6.6)	153 (6.6)
	1-2	68 (7.3)	267 (11.5)
	3-6	355 (38.1)	922 (39.8)
	7 and more	448 (48.0)	974 (42.1)
	Mother listens to radio		
	Almost everyday	60 (6.2)	143 (6.4)
	Not at all/less than once/at least once a week	873 (93.8)	2,172 (93.6)
	Mother reads newspaper		
	Almost everyday	109 (11.8)	536 (23.2)
	Not at all/at least once a week	823 (88.2)	1,779 (76.8)
	Mother listens to television		
	Almost everyday	672 (72.0)	1,926 (83.2)
	Not at all/at least once a week	261 (28.0)	389 (16.8)
	Wealth quintile		
	Poorest	369 (39.6)	380 (16.4)
	Poorer	259 (27.7)	476 (20.6)
	Middle	151 (16.2)	459 (19.8)
	Richer	98 (10.5)	511 (22.1)
	Richest	55 (5.9)	488 (21.1)
Community-level	Class		
	Upper class	349 (48.6)	700 (31.0)
	Scheduled caste	279 (30.8)	451 (19.9)
	Scheduled tribe	32 (3.6)	56 (2.5)
	Other marginalised class	210 (23.2)	934 (41.4)
	State		
	Delhi	99 (10.6)	949 (41.0)
	Maharashtra	625 (67.0)	286 (12.4)
	Tamil Nadu	25 (2.8)	192 (8.3)
	Uttar Pradesh	52 (5.6)	173 (7.5)
	West Bengal	43 (4.6)	166 (7.2)
	Telangana	33 (3.5)	372 (16.1)
	Madhya Pradesh	55 (5.9)	177 (7.7)
Policy level	Place of birth		
	Home	55 (6.0)	177 (7.7)
	Health facility	877 (94.1)	2134 (92.3)

Note: N = number of all participants n = number of participants in each subset, % = percentages.

Source: Authors’ analysis of the 2015–2016 India Demographic and Health Survey (DHS) [24].

A higher proportion of mothers with secondary or higher education lived in urban non-slum areas (77.3%) compared to urban slum areas (68.7%). Most fathers in the urban slum (78.9%) and urban non-slum (87.7%) had no education. Over 90% of mothers in both urban slums and urban non-slums delivered babies at the health facilities. Most mothers watched television almost every day in the urban slum (72%) and urban non-slum areas (83.2%). However, fewer than 20% watched the news or listened to the radio in both areas. In the urban slum areas, only 5.9% of participants belonged to the richest wealth quintile, compared to 21.1% in the urban non-slum areas.

### Breastfeeding practices by socio-demographic characteristics

In urban slum areas, a higher proportion of participants belonged to the upper class (48.6%) compared to other social classes (scheduled castes: 30.8%, marginalized class: 23.2%, scheduled tribes: 3.6%). In contrast, in urban non-slum areas, most participants were from the marginalized class (41.4%) rather than the upper class (31.0%). 67.0% of the participants living in the urban slum were from Maharashtra, and urban non-slum participants were mostly from Delhi (41.0%), compared to the other six states

### Breastfeeding practices across urban slum and urban non-slum areas

**Fig 1:**
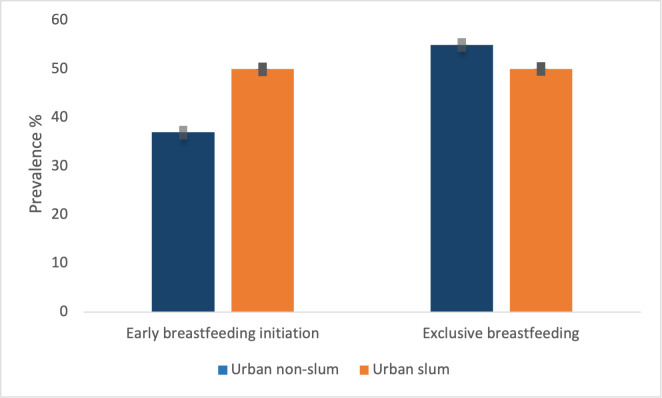
Prevalence of breastfeeding practices stratified by urban slum and urban non-slum areas in India. Error bars represent 95% confidence intervals. Differences between urban slum and urban non-slum areas were assessed using Pearson’s chi-square test.

The prevalence of EBI within an hour was higher in the urban slum (50.4%, 95% CI: 47.2–53.7) than in the urban non-slum areas (37.4%, 95% CI: 35.4–39.4) (Fig 1), but the difference was not statistically significant (p = 0.768). EBF was significantly higher in urban non-slum (55.8%, 95% CI: 51.5–60.0) than in urban slum areas (50.0%, 95% CI: 42.5–57.7) (p = 0.004).

### Multivariable analysis of determinants of breastfeeding practices

At the individual-level in urban slum areas, mothers who reported preceding birth of more than 24 months were less likely to practice early initiation of breastfeeding (adjusted OR: 0.27, 95%CI: 0.10–0.75) compared to mothers who had a preceding birth of less than 24 months (Table 2).

**Table 2:** Correlates of Early Breastfeeding Initiation Practices stratified by Urban slum and urban non-slum areas in India: A multi-level analysis using DHS Data for India, 2015-2016.

		Early Breastfeeding Initiation			
		Urban slum areas		Urban non-slum areas	
	Variables	Crude OR (95% CI)	Adjusted OR (95% CI)	Crude OR (95% CI)	Adjusted OR (95% CI)
Individual-level	Sex				
	Male children	1.00 (ref)		1.00 (ref)	
	Female children	0.89 (0.49-1.61)		0.80 (0.57-1.12)	
	Birth order				
	1^st^	1.00 (ref)		1.00 (ref)	1.00 (ref)
	2^nd^-4^th^	1.15 (0.62-2.11)		1.08 (0.76-1.52)	1.11 (0.77-1.60)
	5^th^ and above	0.20 (0.01-2.78)		1.93 (0.74-5.00)^	2.17 (0.75-6.28)
	Preceding birth interval				
	< 24 months	1.00 (ref)	1.00 (ref)	1.00 (ref)	
	≥24 months	0.45 (0.20-1.01)^	0.27 (0.10-0.75)*	0.80 (0.49-1.31)	
	Antenatal care visits				
	None	1.00 (ref)	1.00 (ref)	1.00 (ref)	
	1-2	0.61 (0.10-3.90)	0.45 (0.05-3.93)	1.20 (0.53-2.70)	
	3-6	1.88 (0.38-3.90)	1.94 (0.31-12.25)	0.88 (0.45-1.74)	
	7 and more	4.36 (0.85-22.26)^	5.80 (0.86-39.13)	1.12 (0.57-2.23)	
	Mother’s Education				
	No education	1.00 (ref)	1.00 (ref)	1.00 (ref)	
	Primary school	1.05 (0.35-3.20)	1.00 (0.26-3.90)	1.41 (0.70-2.84)	
	Secondary and above	1.86 (0.83-4.13)^	1.61 (0.59-4.41)	1.09 (0.66-1.80)	
	Father’s Education				
	No education			1.00 (ref)	
	Primary school			1.78 (0.06-51.35)	
	Secondary and above			0.85 (0.25-1.38)	
	Mother reads radio				
	Not at all/at least once a week	1.00 (ref)		1.00 (ref)	
	Almost every day	1.93 (0.63-5.92)		1.19 (0.62-2.27)	
	Mother reads newspaper				
	Not at all/at least once a week	1.00 (ref)		1.00 (ref)	
	Almost every day	0.91 (0.36-2.34)		0.80 (0.51-1.25)	
	Mother reads tv				
	Not at all/at least once a week	1.00 (ref)	1.00 (ref)	1.00 (ref)	1.00 (ref)
	Almost every day	1.94 (0.91-4.14)^	1.55 (0.62-3.92)	1.18 (0.75-1.87)	
	Wealth quintile				
	Poorest	1.00 (ref)	1.00 (ref)	1.00 (ref)	1.00 (ref)
	Poorer	1.99 (0.82-4.84) ^	2.24 (0.71-7.09)	0.66 (0.36-1.19) ^	0.70 (0.37-1.33)
	Middle	0.79 (0.29-2.13)	0.70 (0.20-2.41)	0.71 (0.40-1.27)	0.74 (0.40-1.37)
	Richer	1.59 (0.56-4.53)	1.22 (0.33-4.53)	0.65 (0.37-1.16) ^	0.63 (0.34-1.17)
	Richest	0.67 (0.19-2.37)	0.51 (0.11-2.41)	0.48 (0.26-0.89) ^	0.47 (0.24-0.90)
Community level factors	Class				
	Upper class	1.00 (ref)	1.00 (ref)	1.00 (ref)	1.00 (ref)
	Scheduled caste	2.02 (0.79-5.16) ^	1.98 (0.68-5.76)	1.34 (0.83-2.16)	1.19 (0.74-1.94)
	Scheduled tribe	3.01 (0.34-26.80)	3.68 (0.35-39.01)	2.82 (0.98-8.15)^	2.41 (0.83-7.02)
	Other marginalized class	0.63 (0.27-1.47)	0.48 (0.19-1.22)	0.95 (0.62-1.46)	0.89 (0.58-1.37)
Policy level	Place of child delivery				
	Home	1.00 (ref)	1.00 (ref)	1.00 (ref)	1.00 (ref)
	Hospital	2.47 (0.88-6.91)^	1.93 (0.56-6.72)	3.21 (1.58-6.52)^	3.80 (1.80-8.02) ***

p-value<0.2^, *p-value<0.05, **p < 0.01, ***p < 0.001 ref-reference, CI-confidence interval, OR-odds ratio.

Due to a lack of variability in the maternal age group (both groups) and the father’s educational level (urban slum), the variable was omitted from the model for that subgroup because of perfect collinearity. This indicates that all mothers in that subgroup fell within the same age category (<20 years).

Model diagnostics: In both the urban slum (Mean VIF = 2.23) and non-slum areas (Mean VIF = 1.59), all VIF values were below the common threshold of 10, indicating no serious multicollinearity among the independent variables.

Random intercepts accounted for clustering at the community level in the urban slum and urban non-slum areas. In the urban slum areas, the intraclass correlation coefficient (ICC) was 0.65, indicating that 65% of the variance in early breastfeeding is attributable to differences between clusters. While in the urban non-slum areas, the ICC was 0.33, indicating that 33% of the variation in early breastfeeding is due to cluster-level differences.

Source: Authors’ analysis of the 2015–2016 India Demographic and Health Survey (DHS) [24].

In the adjusted model for urban non-slum areas, mothers who had their children delivered in the health facilities were more likely to practice early initiation of breastfeeding than mothers who did not deliver their children in health facilities (adjusted OR: 3.80, 95%CI: 1.80–8.02) (Table 2). No statistically significant associations were observed for exclusive breastfeeding (Table 3).

**Table 3:** Correlates of exclusive breastfeeding for children aged less than 6 months old stratified by urban-slum and urban-non slum in India: A multi-level analysis using DHS Data for India, 2015-2016.

		Exclusive breastfeeding for children under 6 months old			
		Urban slum areas		Urban non-slum areas	
	Variables	Crude OR (95% CI)	Adjusted OR (95% CI)	Crude OR (95% CI)	Adjusted OR (95% CI)
Individual-level	Sex				
	Male children	1.00 (ref)		1.00 (ref)	
	Female children	0.57 (0.16-1.99)		0.70 (0.37-1.32)	
	^+^Birth order	0.48 (0.15-1.57)		0.68(0.39-1.17)^	0.93 (0.49-1.77)
	Preceding birth interval				
	< 24 months	1.00 (ref)		1.00 (ref)	
	≥24 months	1.92 (0.30-12.38)		2.62 (0.84-8.18)^	2.03 (0.62-6.62)
	Antenatal care visits				
	None	1.00 (ref)		1.00 (ref)	
	1-2	0.27 (0.00-23.22)		0.51 (0.11-2.41)	
	3-6	7.69 (0.20-302.18)		0.83 (0.24-2.87)	
	7 and more	10.83 (0.23-520.91)		0.80 (0.23-2.78)	
	Mother’s Education				
	No education	1.00 (ref)		1.00 (ref)	
	Primary school	3.76 (0.38-37.21)		2.13 (0.52-8.73)	
	Secondary and above	2.47 (0.49-12.41)		1.39 (0.53-3.69)	
	Father’s Education				
	No education			1.00 (ref)	
	Primary school			#	
	Secondary and above			1.20 (0.30-4.86)	
	Mother reads radio				
	Not at all/at least once a week	1.00 (ref)		1.00 (ref)	
	Almost every day	2.25 (0.16-31.70)		1.77 (0.42-7.54)	
	Mother reads newspaper				
	Not at all/at least once a week	1.00 (ref)		1.00 (ref)	
	Almost every day	1.44 (0.20-10.67)		1.30 (0.53-3.20)	
	Mother reads tv				
	Not at all/at least once a week	1.00 (ref)		1.00 (ref)	
	Almost every day	0.91 (0.25-3.30)		1.12 (0.50-2.52)	
	Wealth quintile				
	Poorest	1.00 (ref)		1.00 (ref)	
	Poorer	0.73 (0.09-6.23)		0.87 (0.28-2.69)	
	Middle	1.18 (0.13-10.99)		1.31 (0.48-3.54)	
	Richer	2.56 (0.22-30.37)		1.31 (0.47-3.68)	
	Richest	2.15 (0.21-21.57)		1.52 (0.53-4.40)	
Community-level	Class				
	Scheduled caste	1.00 (ref)		1.00 (ref)	
	^Scheduled tribe	0.37 (0.09-1.59)		1.17 (0.43-3.20)	
	Marginalized class	#		0.71 (0.07-7.26)	
	Upper class	0.26 (0.06-1.04)		1.00 (0.43-2.32)	
Place of child delivery	Home	1.00 (ref)		1.00 (ref)	1.00 (ref)
	Hospital	3.03 (0.75-12.21)^		3.77 (1.17-12.13)^	3.33 (0.96-11.57)

p-value<0.2^, *p-value<0.05, **p < 0.01, ***p < 0.001, ref-reference category, CI-confidence interval, OR-odds ratio, ^+^Variables were inputted as continuous variables due to perfect collinearity, # no participant in group. Due to a lack of variability in the maternal age group (both groups) and the father’s educational level (urban slum), the variable was omitted from the model for that subgroup because of perfect collinearity. This indicates that all mothers in that subgroup fell within the same age category.

Model diagnostics: In the non-slum areas, all VIF values were below the common threshold of 10, indicating no serious multicollinearity among the independent variables (Mean VIF = 1.17). The variance of the random intercept suggested moderate between-cluster variation. Overall, the model was not statistically significant (Wald χ² = 6.38, p = 0.172).

Source: Authors’ analysis of the 2015–2016 India Demographic and Health Survey (DHS) [24].

## Discussion

In our study, only about half of the mothers living in slum areas initiated breastfeeding within one hour of birth, consistent with findings from a study conducted in an urban slum of Sundar Nagari in East Delhi [28]. In urban non-slum areas, less than half (37%) of the mothers practiced EBI, a rate lower than the national average of 41% [29]. This suggests that challenges to EBI are not limited to urban slum settings alone but may also reflect broader gaps in breastfeeding support within urban health systems. However, the slightly higher EBI rates in slum areas may also indicate targeted program efforts or community-based support initiatives in these settings.

EBF prevalence was lower in urban slums (50%) than in urban non-slum areas (55%). However, other studies have reported even lower rates of EBF in slum settings, which may be partly due to inconsistencies in how EBF indicators are defined [13–15]. According to the WHO definition, which we adopted, the age band for assessing EBF is 0–5 months. In contrast, some studies included six-month-old infants who may have already begun complementary feeding, leading to an underestimation of EBF prevalence [13–15].

Our study revealed correlates of breastfeeding practices at the individual-and policy-levels. At the individual level, a preceding birth interval of more than 24 months in the urban slum was associated with a lower likelihood of practicing EBI compared to a preceding birth interval of less than 24 months. Mothers with shorter intervals may receive more recent counselling or have heightened caution. However, these finding contrasts with evidence by Dwi Tama et al. (2020) that firstborn children had significantly higher odds of delayed breastfeeding initiation compared to the fourth-born child, suggesting that greater birth experience, often equivalent to longer birth intervals that allow mothers to gain skills and confidence, may support earlier breastfeeding initiation [30]. Given the lack of direct comparative evidence, our finding warrants further investigation in similar settings.

At the policy level, our findings show that in non-slum urban areas, mothers who had health facilities for childbirth were more likely to practice early initiation of breastfeeding compared to those born elsewhere. Similar results were reported in a study conducted in the Nagpur slum, India, suggesting that health personnel in facilities play a key role, possibly by promoting breastfeeding through direct counselling or through support provided during childbirth and postpartum care [13,31]. Given that over 90% of mothers in both urban slum and urban non-slum areas delivered in health facilities, the *Mother’s Absolute Affection (MAA)* program [32]—India’s adaptation of the Baby-Friendly Hospital Initiative—should be further strengthened to ensure quality, effective implementation, and inclusion of mothers residing in urban slums.

In India, institutional deliveries are actively promoted through the National Health Mission’s *Janani Suraksha Yojana*(JSY) [33] and *Janani Shishu Suraksha Karyakram* (JSSK) [34] programs, which provide financial incentives and free maternity services to encourage facility-based births. State governments operationalize these initiatives through their respective health departments, supported by community-level mobilization led by Accredited Social Health Activists (ASHAs) [35]. The strong performance of institutional deliveries in our study—exceeding 90% in both urban slum and urban non-slum areas—suggests that these programs have been effective in promoting facility-based births. Leveraging this platform to strengthen post-delivery breastfeeding counselling and follow-up can further improve early initiation and exclusive breastfeeding practices.

The primary limitation of this study is the use of a 24-hour recall to assess children’s feeding practices, which is subject to recall bias. To minimize this bias, the analysis focused on the last-born children living with their mothers. Another limitation is the potential for social desirability bias in responses about breastfeeding practices, especially when incorrect practices are disapproved of within the community. This could lead to an overestimation of correct feeding practices. Additionally, over 80% of data on mothers’ occupations were missing, preventing further analysis of this variable. Nonetheless, we conducted a sensitivity analysis, which showed that excluding this variable did not significantly alter the main findings. Lastly, we were only able to present aggregated breastfeeding prevalence and could not provide state-wise estimates due to small sample sizes.

Despite these limitations, the study has several strengths. To our knowledge, it is the first comparative analysis of breastfeeding practices between urban slums and urban non-slum areas in India using DHS data. The dataset covers six geographical regions of India (Southern, Western, Eastern, Central, Northern, and North-eastern) and uses standardized questionnaires that have been implemented across multiple survey cycles (1998–2015). Moreover, breastfeeding outcomes were assessed according to the WHO standard criteria, enhancing the validity of our findings. Given the wide geographic coverage and standardized data collection, our findings are likely generalizable to urban slum and urban non-slum populations across India and can inform strategies to improve breastfeeding practices.

## Conclusion

Breastfeeding practices were suboptimal in both urban slum and urban non-slum areas in India. The prevalence of EBI was higher in the urban slum than in urban non-slum areas, while EBF prevalence was higher in the urban non-slum than in urban slum areas. Individual- and policy-level factors were associated with breastfeeding practices in India. We recommend policy reforms to leverage health facilities in promoting EBI and EBF. Future researchers should explore the causal pathways of the observed associations in our studies. Lastly, with no associations observed for EBF, future research should examine other contextual factors such as insecurity, marginalization, policies, communicable diseases, and environmental factors like climate-induced droughts and flooding that may play a role in shaping breastfeeding practices.

## Supporting information

S1 File DHS Women Questionnaire. (PDF)
