# Supplementary material for: Prevalence and social determinants of breastfeeding practices in urban slums and urban non-slum areas in India: A comparative analysis
Source: PLoS One. 2026 Apr 8;21(4):e0323861. doi: 10.1371/journal.pone.0323861 (PMC13061255; doi:10.1371/journal.pone.0323861)
Supplement: S1 File — (PDF) [file pone.0323861.s001.pdf]

8 Oct 2015

राष्ट्रीय पररवार स्वास््य सवेक्षण, भारत 2015-16 (NFHS-4)

महिला प्रश्नावली [STATE NAME]

NATIONAL FAMILY HEALTH SURVEY, INDIA 2015-2016 (NFHS-4)

WOMAN'S QUESTIONNAIRE [STATE NAME]

CONFIDENTIAL
For research purposes only

IDENTIFICATION

STATE ______________________

DISTRICT ______________________

TEHSIL/TALUK ______________________

CITY/TOWN/VILLAGE ______________________

TYPE OF PSU (URBAN = 1, RURAL = 2) . . . .

PSU NUMBER . . . .

STRUCTURE NUMBER . . . .

HOUSEHOLD NUMBER . . . .

NAME AND LINE NUMBER OF WOMAN ______________________

ADDRESS OF HOUSEHOLD ______________________

IS HOUSEHOLD SELECTED FOR THE STATE MODULE? (YES = 1, NO = 2) . . . .

IS WOMAN SELECTED FOR QUESTIONS ON HOUSEHOLD RELATIONS (SECTION 11)? (YES = 1, NO = 2) . . . .

INTERVIEWER VISITS

	1	2	3	FINAL VISIT
DATE	______	______	______	DAY<br>MONTH<br>YEAR
INTERVIEWER'S NAME	______	______	______	INT. NO.
RESULT CODE*	______	______	______	RESULT CODE*
NEXT VISIT: DATE<br>TIME	______<br>______	______<br>______		TOTAL NUMBER OF VISITS
SUPERVISOR'S NAME	______			SUPERVISOR NUMBER

*RESULT CODES:

1 COMPLETED
2 NOT AT HOME
3 POSTPONED
4 REFUSED
5 PARTLY COMPLETED
6 INCAPACITATED
7 OTHER ______ (SPECIFY)

**LANGUAGE CODES:

01 ASSAMESE	08 MALAYALAM	15 TAMIL
02 BENGALI	09 MANIPURI	16 TELUGU
03 GUJARATI	10 MARATHI	17 URDU
04 HINDI	11 NEPALI	18 ENGLISH
05 KANNADA	12 ORIYA	19 GARO
06 KASHMIRI	13 PUNJABI	20 KHASI
07 KONKANI	14 SINDHI	96 OTHER ______ SPECIFY

LANGUAGE OF QUESTIONNAIRE **HINDI 0 4

**RESPONDENT'S MOTHER TONGUE ______

**LANGUAGE OF INTERVIEW ______

TRANSLATOR USED? (YES = 1, NO = 2). . . . .

SECTION 1. RESPONDENT'S BACKGROUND

INTRODUCTION AND INFORMED CONSENT

नमस्ते। मेरा नाम _________ िै। मैं(NAME OF ORGANIZATION) केसाथ काम कर रिा/ रिी हूँ। िम पूरेभारत मेंस्वास््य पर एक सवेक्षण कर रिेिैं। जो जानकारी िम पररवार कल्याण और स्वास््य केबारेमेंघरों और व्यहियों सेइकट्ठी करेंगेवो सरकार को स्वास््य सेवाएंबनानेमेंमदद करेगी। आपका पररवार इस सवेक्षण केहलए चुना गया िै। इन सवालों मेंलगभग 40 - 60 हमनट लगेंग। आपकेसारेजवाब गुप्त रखेजायेंग और िमारेसवेक्षण केसदस्यों केअलावा ककसी को भी निीं बतायेजायेंग। आपका इस सवेक्षण मेंभाग लेना स्वैहछिक िैं। अगर आप मेरेककसी सवाल का जवाब निीं देना चािते, तो मुझेबता दीहजयेऔर मैंअगलेसवाल पर चली जाऊँगी या आप ककसी भी समय यि बातचीत रोक सकतेिैं। यकद आपको इस सवेक्षण केबारेमेंऔर जानकारी चाहिए तो आप उस व्यहि को संपकककरें,हजनका कार्कआपकेपररवार को कदया जा चुका िै।

क्या आप मुझसेकुि सवाल पूिना चािती िै?
ANSWER ANY QUESTIONS AND ADDRESS RESPONDENT'S CONCERNS.

क्या आप इस सवेक्षण मेंभाग लेनेकेहलए सिमत िैं?

Namaste. My name is _______. I am working with (NAME OF ORGANIZATION). We are conducting a survey about health all over India. The information on family welfare and health that we collect from households and individuals will help the government to plan health services. Your household was selected for the survey. The questions usually take about 40 - 60 minutes. All of the answers you give will be confidential and will not be shared with anyone other than members of our survey team. Your participation in the survey is voluntary. If I ask you any question you don't want to answer, just let me know and I will go on to the next question or you can stop the interview at any time.

If you have any questions about this survey you may ask me.
GIVE CARD WITH CONTACT INFORMATION.

Do you agree to participate in this survey?

SIGNATURE OF INTERVIEWER: ______________________ DATE: ______________

RESPONDENT AGREES TO BE INTERVIEWED . 1 ↓

RESPONDENT DOES NOT AGREE TO BE INTERVIEWED . 2 → END

NO.	QUESTIONS AND FILTERS	CODING CATEGORIES	SKIP
101	RECORD THE TIME.	HOUR . . . . [ ][ ]<br>MINUTES . . . . [ ][ ]	
102	आपका जन्म ककस मिीनेऔर साल मेंहुआ था?<br>In what month and year were you born?	MONTH . . . . [ ][ ]<br>DON'T KNOW MONTH . . . . 98<br>YEAR . . . . [ ][ ][ ][ ]<br>DON'T KNOW YEAR 9998	
103	हपिलेजन्मकदन पर आपकी आयुककतनी थी?<br>How old were you at your last birthday?<br>COMPARE AND CORRECT 102 AND/OR 103 IF INCONSISTENT.	AGE IN COMPLETED YEARS . [ ][ ]	
104	आप ककतनेसमय सेहनरंतर (CURRENT PLACE OF RESIDENCE) मेंरि रिििैं?<br>How long have you been living continuously in (CURRENT PLACE OF RESIDENCE)?<br>IF LESS THAN 1 YEAR, RECORD '00'	YEARS . . . . [ ][ ]<br>ALWAYS. . . . . 95.....<br>VISITOR . . . . 96.....	
105	क्या आप कभी स्कूल गयी िििैं?<br>Have you ever attended school?	YES . . . . 1.....<br>NO . . . . 2.....	 → 108
106	आपनेकौनसा उछ्चतम दजाकपास ककया िििै?<br>What is the highest standard you completed?	STANDARD. . . . . [ ][ ]	
107	CHECK 106:<br>STANDARD 0-5 [ ] ↓ STANDARD 6 AND ABOVE [ ] →		110
108	अब मैंचाहंगी कक आप मुझेयि वाक्य पढ़कर सुनाएं। SHOW A SENTENCE FROM THE LITERACY CARD TO THE RESPONDENT. IF RESPONDENT CANNOT READ WHOLE SENTENCE,<br>PROBE: क्या आप इस वाक्य केककसी भी भाग को पढ़कर मुझेसुना सकती िििैं?<br>Now I would like you to read this sentence to me. SHOW A SENTENCE FROM THE LITERACY CARD TO THE RESPONDENT.<br>IF RESPONDENT CANNOT READ WHOLE SENTENCE, PROBE: Can you read any part of the sentence to me?	CANNOT READ AT ALL . . . . 1<br>ABLE TO READ ONLY PARTS OF SENTENCE . . . . 2<br>ABLE TO READ WHOLE SENTENCE . 3<br>NO CARD WITH REQUIRED LANGUAGE ____________ 4<br>(SPECIFY LANGUAGE)<br>BLIND/VISUALLY IMPAIRED 5	
109	CHECK 108:<br>CODE '2', '3' OR '4' RECORDED [ ] ↓ CODE '1' OR '5' [ ] → RECORDED		111
110	क्या आप अखबार या पहिका लगभग प्रहतकदन, सप्ताि मेंकम सेकम एक बार, सप्ताि मेंएक बार सेकम या कभी निीं पढ़ती िििैं?<br>Do you read a newspaper or magazine almost every day, at least once a week, less than once a week or not at all?	ALMOST EVERY DAY . . . . 1<br>AT LEAST ONCE A WEEK . . . . 2<br>LESS THAN ONCE A WEEK . . . . 3<br>NOT AT ALL 4	
111	क्या आप रेहर्यो लगभग प्रहतकदन, सप्ताि मेंकम सेकम एक बार, सप्ताि मेंएक बार सेकम या कभी निीं सुनती िििैं?<br>Do you listen to the radio almost every day, at least once a week, less than once a week or not at all?	ALMOST EVERY DAY . . . . 1<br>AT LEAST ONCE A WEEK . . . . 2<br>LESS THAN ONCE A WEEK . . . . 3<br>NOT AT ALL 4	
112	क्या आप टेलीहवजन लगभग प्रहतकदन, सप्ताि मेंकम सेकम एक बार, सप्ताि मेंएक बार सेकम या कभी निीं देखती िििैं?<br>Do you watch television almost every day, at least once a week, less than once a week or not at all?	ALMOST EVERY DAY . . . . 1<br>AT LEAST ONCE A WEEK . . . . 2<br>LESS THAN ONCE A WEEK . . . . 3<br>NOT AT ALL 4	

NO.	QUESTIONS AND FILTERS	CODING CATEGORIES	SKIP
113	क्या आप सामान्यतः मििनेमेंकम सेकम एक बार हसनेमाघर या हथयेटर मेंहसनेमा देखनेजाती ि◌ै◌ं?<br>Do you usually go to a cinema hall or theatre to see a movie at least once a month?	YES . . . . 1<br>NO 2	
114	आपका धमकक्या ि◌ै?<br>What is your religion?	HINDU . . . . 01<br>MUSLIM . . . . 02<br>CHRISTIAN . . . . 03<br>SIKH . . . . 04<br>BUDDHIST/NEO-BUDDHIST . . . . 05<br>JAIN . . . . 06<br>JEWISH . . . . 07<br>PARSI/ZOROASTRIAN . . . . 08<br>NO RELIGION . . . . 09<br>OTHER ______________________ 96<br>(SPECIFY)	
115	आपकी जाहत या जनजाहत क्या ि◌ै?<br>What is your caste or tribe?	CASTE ______________________ 991<br>(SPECIFY)<br>TRIBE ______________________ 992<br>(SPECIFY)<br>NO CASTE/TRIBE . . . . 993<br>DON'T KNOW 998	993 → 201
116	क्या आप अनुसूहचत जाहत, अनुसूहचत जनजाहत, अन्य हपिडेवगकसेि◌ै◌ंया इनमेंसे कोई निीं ि◌ै◌ं?<br>Do you belong to a scheduled caste, a scheduled tribe, other backward class, or none of these?	SCHEDULED CASTE . . . . 1<br>SCHEDULED TRIBE . . . . 2<br>OBC . . . . 3<br>NONE OF THEM 4	

SECTION 2. REPRODUCTION

NO.	QUESTIONS AND FILTERS	CODING CATEGORIES	SKIP
201	अब मैंआपसेउन बच्चों केबारेमेंपूिना चाहूंगी हजन्िेंआपनेअपनेजीवनकाल में जन्म कदया िै। क्या आपनेकभी ककसी बच्चेको जन्म कदया िै?<br><br>Now I would like to ask about all the births you have had during your life. Have you ever given birth?	YES . . . . 1.<br>NO . . . . 2.	 → 206
202	क्या आपकेकोई ऐसेबेटेया बेरटयांिैंहजन्िेंआपनेजन्म कदया िैऔर जो अभी आपके साथ रितेिैं?<br>Do you have any sons or daughters to whom you have given birth who are now living with you?	YES . . . . 1.<br>NO . . . . 2.	 → 204
203	a. ककतनेबेटेआपकेसाथ रितेिैं?<br>How many sons live with you?<br>b. और ककतनी बेरटयांआपकेसाथ रिती िैं?<br>And how many daughters live with you?<br><br>IF NONE, RECORD '00'.	SONS AT HOME . . . . [ ][ ]<br><br>DAUGHTERS AT HOME . . . . . [ ][ ]	
204	क्या आपकेऐसेबेटेया बेरटयांिैंहजन्िेंआपनेजन्म कदया िैऔर जो जीहवत िैंलेककन आपकेसाथ निीं रितेिैं?<br>Do you have any sons or daughters to whom you have given birth who are alive but do not live with you?	YES . . . . 1.....<br>NO . . . . 2.....	 → 206
205	a. ऐसेककतनेजीहवत बेटेिैंजो आपकेसाथ निीं रितेिैं?<br>How many sons are alive but do not live with you?<br>b. और ऐसी ककतनी जीहवत बेरटयांिैंजो आपकेसाथ निीं रिती िैं?<br>And how many daughters are alive but do not live with you?<br><br>IF NONE, RECORD '00'.	SONS ELSEWHERE . . . . [ ][ ]<br><br>DAUGHTERS ELSEWHERE . [ ][ ]	
206	क्या आपनेकभी ककसी लडकेया लडकी को जन्म कदया िैजो जन्म केसमय जीहवत था या थी लेककन बाद मेंहजसकी मृत्युिो गई?<br>IF NO, PROBE: कोई बच्चा जो रोया था या हजसनेजीहवत िोनेका कोई संकेत कदया लेककन बाद मेंजीहवत निीं रिा?<br><br>Have you ever given birth to a boy or girl who was born alive but later died?<br>IF NO, PROBE: Any baby who cried or showed signs of life but did not survive?	YES . . . . 1<br><br>NO 2	 <br>→ 208
207	a. ककतनेलडकों की मृत्युहुई िै?<br>How many boys have died?<br>b. और ककतनी लडककयों की मृत्युहुई िै?<br>And how many girls have died?<br><br>IF NONE, RECORD '00'.	BOYS DEAD . . . . [ ][ ]<br><br>GIRLS DEAD . . . . [ ][ ]	
208	SUM ANSWERS TO 203, 205, AND 207, AND ENTER TOTAL.<br>IF NONE, RECORD '00'.	TOTAL . . . . [ ][ ]	
209	CHECK 208:<br>यि सुहनहित करनेकेहलए कक मैंनेसिी हलखा िै: आपनेअपनेजीवन मेंकुल ______ बच्चों को जन्म कदया िै। क्या यि सिी िै?<br>Just to make sure that I have this right: you have had in TOTAL _____ births during your life. Is that correct?<br>YES [ ] ↓ NO [ ] → PROBE AND CORRECT 201-208 AS NECESSARY.		
210	CHECK 208:<br>ONE OR MORE BIRTHS [ ] ↓ NO BIRTHS [ ] →		226

211 अब मैंआपकेसभी बच्चों केनाम हलखना चाहंगी, चािेवेअभी जीहवत िंैया निीं। शुूआत अपनेसबसेपिलेबच्चेकेजन्म सेकरें।
Now I would like to record the names of all your births, whether still alive or not, starting with the first one you had.
RECORD NAMES OF ALL THE BIRTHS IN 212. RECORD TWINS AND TRIPLETS ON SEPARATE LINES.
(IF THERE ARE MORE THAN 12 BIRTHS, USE AN ADDITIONAL QUESTIONNAIRE STARTING WITH THE SECOND ROW).

212	213	214	215	216	217<br>IF ALIVE:	218<br>IF ALIVE:	219<br>IF ALIVE:	220<br>IF DEAD:	221
आपके (पिले/अगले) बच्चेका नाम क्या रखा गया था?	क्या (NAME) लडका िै या लडकी िै?	क्या इनमे सेकोई जुडवा बच्चेथे?	(NAME) का जन्म ककस मिीनेऔर साल मेंहुआ िैं?<br>PROBE: उसका जन्मकदन क्या था?	क्या (NAME) अभी जीहवत िै?	हपिले जन्मकदन पर (NAME) की आयुककतनी थी?	क्या (NAME) आपकेसाथ रि रिा/रिी िै?	RECORD HOUSE-HOLD LINE NUMBER OF CHILD<br>(RECORD '00' IF CHILD NOT LISTED IN HOUSE-HOLD).	मृत्युकेसमय (NAME) की आयुककतनी थी?<br>IF '1 YR', PROBE:<br>(NAME) उस समय ककतनेमिीनों का था/थी?	क्या (NAME OF PREVIOUS BIRTH) और (NAME) के बीच ककसी दूसरे जीहवत बच्चेका जन्म हुआ था, उन बच्चों को भी शाहमल करें हजनकी जन्म के पिात्मृत्युिो गई िो?
What name was given to your (first/next) baby?<br><br>BIRTH HISTORY NUMBER AND NAME	Is (NAME) a boy or a girl?	Were any of these births twins?	In what month and year was (NAME) born?<br>PROBE: What is his/her birthday?	Is (NAME) still alive?	How old was (NAME) at (his/her) last birthday?<br><br>RECORD AGE IN COM-PLETED YEARS.	Is (NAME) living with you?		How old was (NAME) when he/she died?<br>IF '1 YR', PROBE: How many months old was (NAME)?<br>RECORD DAYS IF LESS THAN 1 MONTH; MONTHS IF LESS THAN TWO YEARS; OR YEARS.	Were there any other live births between (NAME OF PREVIOUS BIRTH) and (NAME), including any children who died after birth?
01	BOY 1<br>GIRL 2	SING 1<br>MULT 2	MONTH<br>YEAR	YES . . 1<br>NO . . . 2<br>220	AGE IN YEARS	YES . . . 1<br>NO 2	LINE NUMBER<br>(NEXT BIRTH)	DAYS . . . 1<br>MONTHS 2<br>YEARS . . 3	
02	BOY 1<br>GIRL 2	SING 1<br>MULT 2	MONTH<br>YEAR	YES . . 1<br>NO . . . 2<br>220	AGE IN YEARS	YES . . . 1<br>NO 2	LINE NUMBER<br>(GO TO 221)	DAYS . . . 1<br>MONTHS 2<br>YEARS . . 3	YES . . . . 1<br>ADD BIRTH<br>NO . . . . . 2<br>NEXT BIRTH
03	BOY 1<br>GIRL 2	SING 1<br>MULT 2	MONTH<br>YEAR	YES . . 1<br>NO . . . 2<br>220	AGE IN YEARS	YES . . . 1<br>NO 2	LINE NUMBER<br>(GO TO 221)	DAYS . . . 1<br>MONTHS 2<br>YEARS . . 3	YES . . . . 1<br>ADD BIRTH<br>NO . . . . . 2<br>NEXT BIRTH
04	BOY 1<br>GIRL 2	SING 1<br>MULT 2	MONTH<br>YEAR	YES . . 1<br>NO . . . 2<br>220	AGE IN YEARS	YES . . . 1<br>NO 2	LINE NUMBER<br>(GO TO 221)	DAYS . . . 1<br>MONTHS 2<br>YEARS . . 3	YES . . . . 1<br>ADD BIRTH<br>NO . . . . . 2<br>NEXT BIRTH
05	BOY 1<br>GIRL 2	SING 1<br>MULT 2	MONTH<br>YEAR	YES . . 1<br>NO . . . 2<br>220	AGE IN YEARS	YES . . . 1<br>NO 2	LINE NUMBER<br>(GO TO 221)	DAYS . . . 1<br>MONTHS 2<br>YEARS . . 3	YES . . . . 1<br>ADD BIRTH<br>NO . . . . . 2<br>NEXT BIRTH
06	BOY 1<br>GIRL 2	SING 1<br>MULT 2	MONTH<br>YEAR	YES . . 1<br>NO . . . 2<br>220	AGE IN YEARS	YES . . . 1<br>NO 2	LINE NUMBER<br>(GO TO 221)	DAYS . . . 1<br>MONTHS 2<br>YEARS . . 3	YES . . . . 1<br>ADD BIRTH<br>NO . . . . . 2<br>NEXT BIRTH
07	BOY 1<br>GIRL 2	SING 1<br>MULT 2	MONTH<br>YEAR	YES . . 1<br>NO . . . 2<br>220	AGE IN YEARS	YES . . . 1<br>NO 2	LINE NUMBER<br>(GO TO 221)	DAYS . . . 1<br>MONTHS 2<br>YEARS . . 3	YES . . . . 1<br>ADD BIRTH<br>NO . . . . . 2<br>NEXT BIRTH

212	213	214	215	216	217<br>IF ALIVE:	218<br>IF ALIVE:	219<br>IF ALIVE:	220<br>IF DEAD:	221
आपके<br>(पिले/अगले)<br>बछ्चेका नाम<br>क्या रखा गया<br>था?	क्या<br>(NAME)<br>लडका िै<br>या लडकी<br>िै?	क्या इनमे<br>सेकोई<br>जुडवा<br>बछ्चेथे?	(NAME) का जन्म<br>ककस मिीनेऔर साल<br>मेंहुआ िैं?<br>PROBE: उसका<br>जन्मकदन क्या था?	क्या<br>(NAME)<br>अभी<br>जीहवत<br>िै?	हपिले<br>जन्मकदन पर<br>(NAME) की<br>आयुककतनी<br>थी?	क्या<br>(NAME)<br>आपकेसाथ<br>रि<br>रिा/रिी<br>िै?	RECORD<br>HOUSE-<br>HOLD LINE<br>NUMBER<br>OF CHILD<br>(RECORD<br>'00' IF CHILD<br>NOT LISTED<br>IN HOUSE-<br>HOLD).	मृत्युकेसमय (NAME)<br>की आयुककतनी थी?<br>IF '1 YR', PROBE:<br>(NAME) उस समय<br>ककतनेमिीनों का था/थी?	क्या (NAME<br>OF<br>PREVIOUS<br>BIRTH) और<br>(NAME) के<br>बीच ककसी दूसरे<br>जीहवत बछ्चेका<br>जन्म हुआ था,<br>उन बछ्चों को भी<br>शाहमल करें<br>हजनकी जन्म के<br>पिात्मुत्युिो<br>गई िो?
What name was given to your (first/next) baby?<br>BIRTH HISTORY NUMBER AND NAME	Is (NAME) a boy or a girl?	Were any of these births twins?	In what month and year was (NAME) born?<br>PROBE: What is his/her birthday?	Is (NAME) still alive?	How old was (NAME) at (his/her) last birthday?<br>RECORD AGE IN COM-PLETED YEARS.	Is (NAME) living with you?		How old was (NAME) when he/she died?<br>IF '1 YR', PROBE: How many months old was (NAME)?<br>RECORD DAYS IF LESS THAN 1 MONTH; MONTHS IF LESS THAN TWO YEARS; OR YEARS.	Were there any other live births between (NAME OF PREVIOUS BIRTH) and (NAME), including any children who died after birth?
08	BOY 1<br>GIRL 2	SING 1<br>MULT 2	MONTH<br>YEAR	YES . . 1<br>NO . . . 2<br>↓ 220	AGE IN YEARS	YES . . . 1<br>NO 2	LINE NUMBER<br>↓<br>(GO TO 221)	DAYS . . . 1<br>MONTHS 2<br>YEARS . . 3	YES . . . . 1<br>ADD BIRTH<br>NO . . . . . 2<br>NEXT BIRTH
09	BOY 1<br>GIRL 2	SING 1<br>MULT 2	MONTH<br>YEAR	YES . . 1<br>NO . . . 2<br>↓ 220	AGE IN YEARS	YES . . . 1<br>NO 2	LINE NUMBER<br>↓<br>(GO TO 221)	DAYS . . . 1<br>MONTHS 2<br>YEARS . . 3	YES . . . . 1<br>ADD BIRTH<br>NO . . . . . 2<br>NEXT BIRTH
10	BOY 1<br>GIRL 2	SING 1<br>MULT 2	MONTH<br>YEAR	YES . . 1<br>NO . . . 2<br>↓ 220	AGE IN YEARS	YES . . . 1<br>NO 2	LINE NUMBER<br>↓<br>(GO TO 221)	DAYS . . . 1<br>MONTHS 2<br>YEARS . . 3	YES . . . . 1<br>ADD BIRTH<br>NO . . . . . 2<br>NEXT BIRTH
11	BOY 1<br>GIRL 2	SING 1<br>MULT 2	MONTH<br>YEAR	YES . . 1<br>NO . . . 2<br>↓ 220	AGE IN YEARS	YES . . . 1<br>NO 2	LINE NUMBER<br>↓<br>(GO TO 221)	DAYS . . . 1<br>MONTHS 2<br>YEARS . . 3	YES . . . . 1<br>ADD BIRTH<br>NO . . . . . 2<br>NEXT BIRTH
12	BOY 1<br>GIRL 2	SING 1<br>MULT 2	MONTH<br>YEAR	YES . . 1<br>NO . . . 2<br>↓ 220	AGE IN YEARS	YES . . . 1<br>NO 2	LINE NUMBER<br>↓<br>(GO TO 221)	DAYS . . . 1<br>MONTHS 2<br>YEARS . . 3	YES . . . . 1<br>ADD BIRTH<br>NO . . . . . 2<br>NEXT BIRTH

NO.	QUESTIONS AND FILTERS	LAST BIRTH<br>NAME ______	NEXT-TO-LAST BIRTH<br>NAME ______	SECOND-FROM-LAST BIRTH<br>NAME ______
478	CHECK 226:<br>IS RESPONDENT PREGNANT?	NOT PREGNANT ☐ ↓<br>PREGNANT OR UNSURE ☐ (SKIP TO 480)		
479	(NAME) केजन्म केबाद, क्या आपने शारीररक संबंध शुू ककए िैं?<br>Have you had sexual relations since the birth of (NAME)?	YES . . . . 1<br>NO . . . . 2<br>(SKIP TO 481)←		
480	(NAME) केजन्म केबाद, आपनेककतने मिीनों तक शारीररक संबंध निीं रखे?<br>For how many months after the birth of (NAME) did you not have sexual relations?	MONTHS . . . ☐☐<br>DON'T KNOW . . . . . 98.	MONTHS . . . ☐☐<br>DON'T KNOW 98	MONTHS . . . . ☐☐<br>DON'T KNOW 98
481	क्या (NAME) को आपनेकभी स्तनपान कराया?<br>Did you ever breastfeed (NAME)?	YES . . . . 1..<br>NO . . . . 2..<br>(SKIP TO 488)←	YES . . . . 1<br>NO . . . . 2<br>(SKIP TO 488)←	YES . . . . 1<br>NO . . . . 2<br>(SKIP TO 488)←
482	जन्म केककतनेसमय बाद आपने(NAME) को पिली बार स्तनपान कराया था?<br>How long after birth did you first put (NAME) to the breast?<br>IF LESS THAN ONE HOUR, RECORD '00' HOURS.<br>IF LESS THAN 24 HOURS, RECORD HOURS.<br>OTHERWISE, RECORD DAYS.	IMMEDIATELY . . . . 000<br>HOURS . 1 ☐☐<br>DAYS . . . 2 ☐☐		
483	जन्म केबाद केपिलेतीन कदनों में,क्या (NAME) को माूँकेदूध केअलावा, कुि और पीनेको कदया गया था?<br>In the first three days after delivery, was (NAME) given anything to drink other than breast milk?	YES . . . . 1<br>NO . . . . 2<br>(SKIP TO 485)←		
484	(NAME) को पीनेकेहलए क्या कदया गया था?<br>What was (NAME) given to drink? Anything else?<br>RECORD ALL LIQUIDS MENTIONED.	MILK (OTHER THAN BREAST MILK). A<br>PLAIN WATER . . . . . . B<br>SUGAR OR GLUCOSE WATER . . . . C<br>GRIPE WATER . . . . . . D<br>SUGAR-SALT-WATER SOLUTION . . . . . . E<br>FRUIT JUICE . . . . . . F<br>INFANT FORMULA . G<br>TEA . . . . H<br>HONEY. . . . . I<br>JANAM GHUTTI . . . . J<br>OTHER ______ X<br>(SPECIFY)		
485	CHECK 404:<br>IS CHILD LIVING?	LIVING ☐ ↓<br>DEAD ☐ (SKIP TO 487)		

NO.	QUESTIONS AND FILTERS	LAST BIRTH<br>NAME ______	NEXT-TO-LAST BIRTH<br>NAME ______	SECOND-FROM-LAST BIRTH<br>NAME ______
486	क्या आप (NAME) को अभी भी स्तनपान करा रिी िैं?<br>Are you still breastfeeding (NAME)?	YES . . . . .1<br>(SKIP TO 489)<br>NO 2		
487	आपने(NAME) को ककतनेमिीनों तक स्तनपान कराया?<br>For how many months did you breastfeed (NAME)?	MONTHS . . . [ ][ ]<br>DON'T KNOW 98		
488	CHECK 404:<br><br>IS CHILD LIVING?	LIVING [ ] ↓<br>DEAD [ ] (GO BACK TO 405 IN NEXT COLUMN; OR, IF NO MORE BIRTHS, GO TO 491)	LIVING [ ] ↓<br>DEAD [ ] (GO BACK TO 405 IN NEXT COLUMN; OR, IF NO MORE BIRTHS, GO TO 491)	LIVING [ ] ↓<br>DEAD [ ] (GO BACK TO 405 IN NEXT-TO-LAST COLUMN OF NEW QUESTIONNAIRE; OR, IF NO MORE BIRTHS, GO TO 491)
489	क्या (NAME) नेकल या हपिली रात को हनप्पल वाली बोतल सेकुि हपया था?<br>Did (NAME) drink anything from a bottle with a nipple yesterday or last night?	YES . . . . .1<br>NO . . . . .2<br>DON'T KNOW . . . 8	YES . . . . 1<br>NO . . . . 2<br>DON'T KNOW 8	YES . . . . 1<br>NO . . . . 2<br>DON'T KNOW 8
490		GO BACK TO 405 IN NEXT COLUMN; OR, IF NO MORE BIRTHS, GO TO 491.	GO BACK TO 405 IN NEXT COLUMN; OR, IF NO MORE BIRTHS, GO TO 491.	GO BACK TO 405 IN NEXT-TO-LAST COLUMN OF NEW QUESTIONNAIRE; OR, IF NO MORE BIRTHS, GO TO 491.

NO.	QUESTIONS AND FILTERS	CODING CATEGORIES	SKIP
491	CHECK 215 AND 218:<br><br>HAS AT LEAST ONE CHILD BORN IN 2014 OR LATER AND LIVING WITH HER ☐<br>RECORD NAME OF YOUNGEST CHILD LIVING WITH HER (AND CONTINUE WITH 492)<br><br>(NAME) ________________	DOES NOT HAVE ANY CHILDREN BORN IN 2014 OR LATER AND LIVING WITH HER ☐	501

492 अब मैंतरल पदार्थों और खाद्य पदार्थों केबारेमेंपूिना चाहूँगी जो (NAME FROM 491) नेकल कदन मेंया रात मेंहलया/ली था/थी। अगर ये पदाथकआपकेबच्चेनेककसी अन्य आिार मेंहमलाकर भी हलया/ली िो तो भी मैंजानना चाहूँगी ।

क्या (NAME FROM 491) ने _______________ (खाया / पीया) ।

Now I would like to ask you about liquids or foods that (NAME FROM 491) had yesterday during the day or at night. I am interested in whether your child had the item I mention even if it was combined with other foods.

Did (NAME FROM 491) (drink/eat):

		YES	NO	DK
a. सादा पानी?<br>Plain water?	a.	1	2	8
b. जूस या जूस पेय?<br>Juice or juice drinks?	b.	1	2	8
c. सादा सूप?<br>Clear broth?	c.	1	2	8
d. दूध जैसेहर्ब्बा-बंदी, पावर्र या जानवर का ताजा दूध?<br>IF YES: (NAME) को ककतनेबार ऐसा दूध कदया गया?<br>Milk such as tinned, powdered, or fresh animal milk?<br>IF YES: How many times did (NAME) drink milk?<br>IF 7 OR MORE TIMES, RECORD '7'.	d.	1	2	8
	NUMBER OF TIMES DRANK MILK ☐			
e. व्यापाररक ूप सेउत्पाकदत हशशुुस्खा?<br>IF YES: (NAME) को ककतनेबार ye कदया गया?<br>Infant formula?<br>IF YES: How many times did (NAME) drink infant formula?<br>IF 7 OR MORE TIMES, RECORD '7'.	e.	1	2	8
	NUMBER OF TIMES DRANK FORMULA ☐			
f. कोई और तरल पदाथक?<br>Any other liquids?	f.	1	2	8
g. दिी?<br>IF YES: (NAME) को ककतनेबार दिी कदया गया?<br>Yogurt?<br>IF YES: How many times did (NAME) eat yogurt?<br>IF 7 OR MORE TIMES, RECORD '7'.	g.	1	2	8
	NUMBER OF TIMES ATE YOGURT ☐			
h. व्यापाररक ूप सेउत्पाकदत बेबी फ़ूर् जैसेसेरेलैक्स या फॅरेक्स?<br>Any commercially fortified baby food, e.g. Cerelac or Farex?	h.	1	2	8
i. कोई ब्रेलरोटी, रोटी, चपाती, चावल, नूर्ल्स, हबहस्कट, इर्ली या अन्य कोई अनाज सेबना हुआ खाद्यपदाथक?<br>Any bread, roti, chapati, rice, noodles, biscuits, idli, or any other foods made from grains?	i.	1	2	8
j. कोई कद्दू, गाजर, सकरकंद या मीठेआलूजो अंदर सेपीलेया नारंगी रंगके िैं?<br>Any pumpkin, carrots, squash or sweet potatoes that are yellow or orange inside?	j.	1	2	8
k. आलू, सुरन, अरबी, रतालूया अन्य कोई कंदमूल खाद्यपदाथकजो जड सेबनेिो?<br>Any white potatoes, white yams, manioc, cassava, or any other foods made from roots?	k.	1	2	8
l. कोई गिरी िरी पत्तेदार साग (सब्जी)?<br>Any dark green, leafy vegetables?	l.	1	2	8
m. पका हुआ आम, पपीता, खरबूजा या कटिल?<br>Any ripe mangoes, papayas, cantaloupe or jackfruit?	m.	1	2	8

NO.	QUESTIONS AND FILTERS	CODING CATEGORIES	SKIP
	n. कोई अन्य फल या सहब्जयां?<br>Any other fruits or vegetables?	n. 1 2 8	
	o. कोई कलेजी, गुरदा, कदल या कोई दूसरेभाग का गोश्त?<br>Any liver, kidney, heart or other organ meat?	o. 1 2 8	
	p. कोई मुगाक, बत्तख या अन्य पक्षी?<br>Any chickens, duck, or other birds?	p. 1 2 8	
	q. ककसी अन्य प्रकार का गोश्त?<br>Any other meat?	q. 1 2 8	
	r. कोई अंर्े?<br>Any eggs?	r. 1 2 8	
	s. ताजी या सूखी मिली या कर्ेखोलवाली मिहलयाँ या अन्य समुरी जीव जैसेकेकडे?<br>Any fresh or dried fish or shellfish?	s. 1 2 8	
	t. फहलयाँ,सोयाहबन,चना,मटर,राजमा या दालों सेतैयार ककए गए कोई खाद्य पदाथक?<br>Any foods made from beans, peas, lentils, or nuts?	t. 1 2 8	
	u. पनीर या अन्य दूध सेबनेखाद्य पदाथक?<br>Any cheese or other food made from milk?	u. 1 2 8	
	v. कोई अन्य ठोस, अधकठोस या नरम खाद्य पदाथक?<br>Any other solid, semi-solid, or soft food?	v. 1 2 8	
493	CHECK 492 CATEGORIES 'g' THROUGH 'v':<br>NOT A SINGLE 'YES' ☐ ↓	AT LEAST ONE 'YES' ☐ →	495
494	कल कदन मेंया रात मेंक्या (NAME) नेकोई ठोस, अधकठोस या नरम आिार खाया था?<br>IF 'YES' PROBE: ककस तरि केठोस, अधकठोस या नरम आिार (NAME) ने खाए थे?<br>Did (NAME) eat any solid, semi-solid, or soft foods yesterday during the day or at night? IF 'YES' PROBE: What kind of solid, semi-solid or soft foods did (NAME) eat?	YES . . . . 1.<br>(GO BACK TO 492 TO RECORD FOOD EATEN YESTERDAY)<br>NO . . . . 2.	 <br><br>501
495	कल कदन मेंया रात में(NAME) नेककतनी बार कोई ठोस, अधकठोस या नरम आिार खाया था/थी?<br>How many times did (NAME) eat solid, semi-solid, or soft foods yesterday during the day or at night?<br>IF 7 OR MORE TIMES, RECORD '7'.	NUMBER OF TIMES . . . . ☐<br>DON'T KNOW . . . . 8.	

NO.	QUESTIONS AND FILTERS	CODING CATEGORIES	SKIP
911	यद्यहप आपनेहपिलेसात कदनों मेंकाम निीं ककया तो भी क्या आपकेपास कोई नौकरी या व्यापार िैहजससेआप िुट्टी, बीमारी, अवकाश, प्रसूहत िुट्टी या ककसी अन्य ऐसेकारण सेअनुपहस्थत थीं?<br>Although you did not work in the last seven days, do you have any job or business from which you were absent for leave, illness, vacation, maternity leave or any other such reason?	YES . . . . 1<br>NO 2	1 → 913
912	हपिले12 मिीनों मेंक्या आपनेकोई काम ककया िै?<br>Have you done any work in the last 12 months?	YES . . . . 1<br>NO 2	2 → 917
913	आपका व्यवयास क्या िै, अथाकत्मुख्यतः आप ककस प्रकार का काम करती िैं?<br>What is your occupation, that is, what kind of work do you mainly do?	______________________ [ ][ ]	
914	क्या आप यि काम अपनेपररवार केसदस्य केहलए या ककसी अन्य केहलए करती िैंया आपका खुद का व्यवसाय िै?<br>Do you do this work for a member of your family, for someone else, or are you self-employed?	FOR FAMILY MEMBER . . . . . 1<br>FOR SOMEONE ELSE . . . . . 2<br>SELF-EMPLOYED 3	
915	क्या आप सामान्यतः पूरेवषकमेंकाम करती िैं,ककसी हवशेष मौसम मेंकाम करती िैं या केवल कभी-कभार िी काम करती िैं?<br>Do you usually work throughout the year, or do you work seasonally, or only once in a while?	THROUGHOUT THE YEAR . . . . . 1<br>SEASONALLY/PART OF THE YEAR . . . 2<br>ONCE IN A WHILE 3	
916	इस काम केहलए क्या आपको नगद भुगतान ककया जाता िैया कोई वस्तुहमलती िै, या कुि भी निीं कदया जाता िै?<br>Are you paid in cash or kind for this work, or are you not paid at all?	CASH ONLY . . . . . 1<br>CASH AND KIND . . . . . 2<br>IN KIND ONLY . . . . . 3<br>NOT PAID 4	
917	हपिले12 मिीनों मेंक्या आप लगातार एक मिीनेया उससेअहधक समय केहलए घर (माता-हपता/सास-ससुर केघर केअहतररक्त) सेदूर रिी िै?<br>In the last 12 months, have you been away from home other than parental/in-laws home for one month or more at a time?	YES . . . . 1<br>NO 2	2 → 919
918	हपिले12 मिीनों मेंक्या आप लगातार 6 मिीनेया उससेअहधक समय केहलए घर (माता-हपता/सास-ससुर केघर केअहतररक्त) सेदूर रिी िै?<br>In the last 12 months, have you been away from home other than parental/in-laws home for six months or more at a time?	YES . . . . 1<br>NO 2	
919	CHECK 301: MARITAL STATUS<br>CURRENTLY MARRIED [ ] ↓ OTHER [ ] →		927
920	CHECK 916: CASH EARNINGS<br>CODE '1' OR '2' CIRCLED [ ] ↓ OTHER [ ] →		923
921	आपकेद्वारा कमाए गए ुपये-पैसों का उपयोग ककस तरि ककया जाए इसका हनणकय कौन करता िैः मुख्यतः आप, मुख्यतः आपकेपहत या आप और आपकेपहत हमलकर?<br>Who decides how the money you earn will be used: mainly you, mainly your husband, or you and your husband jointly?	RESPONDENT . . . . . 1<br>HUSBAND . . . . . 2<br>RESPONDENT AND HUSBAND JOINTLY . . . . . 3<br>OTHER 6	
922	क्या आप यि किंगी कक आप जो ुपये-पैसेकमाती िैंवि आपकेपहत जो कमातेिैं उससेअहधक िै, कम िैया लगभग उतना िी िै?<br>Would you say that the money that you earn is more than what your husband earns, less than what he earns, or about the same?	MORE THAN HUSBAND . . . . . 1<br>LESS THAN HUSBAND . . . . . 2<br>ABOUT THE SAME . . . . . 3<br>HUSBAND HAS NO EARNINGS . . . . . 4<br>DON'T KNOW 8	4 → 924

NO.	QUESTIONS AND FILTERS	CODING CATEGORIES	SKIP
923	आपकेपहत द्वारा कमाए गए ुपये-पैसों का उपयोग ककस तरि ककया जाए इसका हनणकय कौन करता िैिै: मुख्यतः आप, मुख्यतः आपकेपहत या आप और आपकेपहत हमलकर?<br>Who decides how your husband's earnings will be used: mainly you, mainly your husband, or you and your husband jointly?	RESPONDENT . . . . 1<br>HUSBAND . . . . 2<br>RESPONDENT AND<br>HUSBAND JOINTLY . . . . 3<br>HUSBAND HAS<br>NO EARNINGS . . . . 4<br>OTHER 6	
924	आपकेअपनेस्वास्थ की देखभाल केबारेमेंआमतौर पर कौन हनणकय लेता िैिैं:मुख्यतः आप, मुख्यतः आपकेपहत, आप और आपकेपहत हमलकर या और कोई?<br>Who usually makes decisions about health care for yourself: mainly you, mainly your husband, you and your husband jointly, or someone else?	RESPONDENT . . . . 1<br>HUSBAND . . . . 2<br>RESPONDENT AND<br>HUSBAND JOINTLY . . . . 3<br>SOMEONE ELSE . . . . 4<br>OTHER 6	
925	घर केर्मिंगेंसामानों की खरीददारी केबारेमेंहनणकय आमतौर पर कौन लेता िैिै: मुख्यतः आप, मुख्यतः आपकेपहत, आप और आपकेपहत हमलकर या और कोई?<br>Who usually makes decisions about making major household purchases: mainly you, mainly your husband, you and your husband jointly, or someone else?	RESPONDENT . . . . 1<br>HUSBAND . . . . 2<br>RESPONDENT AND<br>HUSBAND JOINTLY . . . . 3<br>SOMEONE ELSE . . . . 4<br>OTHER 6	
926	आपकेमायकेकेपररवार या ररश्तेदारों केपास जानेकेबारेमेंआमतौर पर कौन हनणकय लेता िैिैं:मुख्यतः आप, मुख्यतः आपकेपहत, आप और आपकेपहत हमलकर या और कोई?<br>Who usually makes decisions about visits to your family or relatives: mainly you, mainly your husband, you and your husband jointly, or someone else?	RESPONDENT . . . . 1<br>HUSBAND . . . . 2<br>RESPONDENT AND<br>HUSBAND JOINTLY . . . . 3<br>SOMEONE ELSE . . . . 4<br>OTHER 6	
927	क्या आपकेअपनेपास कोई ूपया-पैसा िैिैहजसका उपयोग कैसेकरना िैिैइस बारेमें आप अकेलेहनणकय लेसकती िैिैं?<br>Do you have any money of your own that you alone can decide how to use?	YES . . . . 1<br>NO 2	
928	क्या आपको इन स्थानों पर सामान्यतः अकेलेजानेकी या केवल ककसी केसाथ जानेकी अनुमहत िैिै, या हबल्कुल अनुमहत निीं िैिै?<br>Are you usually allowed to go to the following places alone, only with someone else, or not at all?<br>a. बाजार में?<br>To the market?<br>b. स्वास््य सुहवधा में?<br>To the health facility?<br>c. (गांव/समुदाय) केबािर केस्थान पर?<br>To places outside this (village/community)?	ALONE / WITH SOMEONE ELSE ONLY / NOT AT ALL<br>MKT . . . . 1 2 3<br>HEALTH . . . . . 1 2 3<br>OUT 1 2 3	
929	क्या आपकेपास बैंक या बचत खाता िैिैहजसका आप खुद इस्तेमाल करती िैिैं?<br>Do you have a bank or savings account that you yourself use?	YES . . . . 1<br>NO 2	
930	क्या आपकेपास कोई अपना मोबाइल फोन िैिै, हजसका उपयोग आप ख़ुद करती िैिै?<br>Do you have any mobile phone that you yourself use?	YES . . . . 1<br>NO 2	→ 931
930A	CHECK 106: EDUCATION<br>STANDARD 0-5 OR BLANK ☐ ↓	STANDARD 6 AND ABOVE ☐	→ 930C
930B	CHECK 108: LITERACY<br>CODE '2', '3' OR '4' CIRCLED ☐ ↓	CODE '1' OR '5' CIRCLED ☐	→ 931
930C	क्या आप हलखा हुआ संदेश (SMS) पढ़ सकतेिैिैं?<br>Are you able to read text (SMS) messages?	YES . . . . 1<br>NO 2	

INTERVIEWER'S OBSERVATIONS

TO BE FILLED IN AFTER COMPLETING INTERVIEW

COMMENTS ABOUT RESPONDENT:

COMMENTS ON SPECIFIC QUESTIONS:

ANY OTHER COMMENTS:

SUPERVISOR'S OBSERVATIONS

NAME OF SUPERVISOR: ____________________ DATE: ____________________
